# Shift workers’ perceptions and experiences of adhering to a nutrition intervention at night whilst working: a qualitative study

**DOI:** 10.1038/s41598-022-19582-x

**Published:** 2022-09-15

**Authors:** Catherine E. Huggins, Jessica Jong, Gloria K. W. Leung, Sophie Page, Rochelle Davis, Maxine P. Bonham

**Affiliations:** Department of Nutrition, Dietetics and Food, Monash University, Level 1 264 Ferntree Gully Rd, Notting Hill, VIC 3168 Australia

**Keywords:** Health occupations, Risk factors

## Abstract

This study explored the feasibility of implementing a meal timing intervention during night shift work. Data were collected via semi-structured interviews. Interviews were coded inductively by two researchers independently, then three major themes were collaboratively developed. Subthemes from each major theme were mapped to the theoretical domains framework and the Capability Opportunity Motivation model of behaviour change. Seventeen night shift workers (rotating or permanent) aged between 25 and 65 years were interviewed. Participants predominately worked as health professionals. The feasibility of a simple meal timing intervention to avoid eating between 1 and 6 am on night shift is largely affected by three major influences (1) physical and emotional burden of shift work which drives food temptations; (2) the workplace context including the meal break environment, social and cultural context at work, and break scheduling; and (3) motivation of the individual. Facilitators to avoiding eating at night were, keeping busy, having co-worker support, management support, education of health benefits and/or belief that the intervention was health promoting. The barriers to avoiding eating at night were the emotional and physical toll of working at night leading to comfort eating and not having rest areas away from food environments. To support night shift workers with changing timing of meals, interventions at work should target both individual and organisational level behaviour change.

## Introduction

Shift workers make up 16% to 20% of the workforce in Australia, America and the European Union^1–3^ and health care workers represent a quarter of these workers^1^. Working shift work disproportionately increases risk of chronic disease due to atypical work schedules^4^. A meta-analysis of observational studies found that overall, exposure to any kind of shift work was associated with 9% increased odds of developing Type 2 diabetes, while individuals who rotate through both day and night shifts face 42% increased odds compared to their day-working counterparts^5^. Shift workers have a 23% increased risk of myocardial infarction and 5% increased risk of stroke compared to non-shift workers^6^. Despite having the same daily energy intake as day workers^7^, shift workers have a 23% increased risk of developing overweight/obesity and 35% increased risk of developing abdominal obesity compared to day workers^8^.

Humans are diurnal animals, therefore food intake is favoured during the day. The endogenous circadian clock system regulates a number of physiological processes, such as digestion, glucose metabolism and insulin production, ensuring that they occur at appropriate times of the 24-h day^9,10^. Shift work disrupts circadian and behavioural rhythms, shifting food intake to during the night when working night shifts. Studies in day workers and simulated shift work conditions have demonstrated elevated postprandial glucose, insulin and triglycerides responses following consumption of food at night^11–16^, which if experienced over a long time, may be risks for development of cardiovascular disease and type 2 diabetes. Experimental studies have demonstrated the long term metabolic disadvantages of eating during the ‘biological night’; nocturnal mice fed a high fat diet during the day over a 6-week period gained significantly more weight than mice fed during the night^17^.

Factors influencing shift workers’ dietary behaviours, dietary patterns and food choices have been explored previously and are shown to be affected by a range of factors including physiological, psychosocial, environmental, and organisational influences^18–20^. It is plausible that shift workers’ risk of chronic diseases may be attenuated by restricting overnight food intake through an improvement in glucose and lipid regulation, but this is yet to be investigated.

We undertook a pilot meal timing intervention study as a first step towards testing the hypothesis that avoiding eating at night will improve glucose and lipid regulation of night shift workers (rotating or permanent night shifts). Participants were required to change their usual eating times so that they avoided eating from 1 to 6 am. This was considered a simple intervention as it did not seek to change the amount or type of foods eaten. The objective of the present study was to explore the experiences of night shift workers participating in this pilot intervention study to understand the feasibility of implementing a meal timing intervention during night shift work. Overall, there has been very little published in the literature examining the feasibility of dietary interventions designed to improve health outcomes in night shift workers^21^. The results of this exploration are then discussed in the context of the Theoretical Domains Framework^22^ to guide future intervention design and implementation.

## Methods

### Participants and intervention description

All participants enrolled in the intervention study were eligible and invited to participate in this qualitative exploration. The intervention study was a randomised crossover trial (Trial registered 30/05/2017 ACTRN12617000791336) which is reported elsewhere^23^ and the qualitative interviews were conducted after each participant completed the trial. All data collection and thematic analysis occurred between July 2017 and October 2018. Participants were night shift workers (rotating or permanent) with abdominal obesity. Eligible participants were permanent or rotating night shift workers, involved in night shift work for a minimum of 12 months consecutively prior to study commencement and were expecting to work at least three night shifts per fortnight during the study’s duration. Participation involved four weeks of overnight food avoidance (intervention) and four weeks of habitual dietary pattern maintenance (control), separated by a 2-week washout period. The details of the intervention study protocol have been published^23^. During the intervention period, participants were instructed to avoid food and beverage consumption (water was permitted) from 1 to 6 am, and redistribute their energy consumption so that overall, they maintained their usual daily energy intake. Prior to beginning the intervention, all participants had a consultation with an Accredited Practising Dietitian, who assisted them in rearranging their meal times around 1 am to 6 am. At completion of the study, participants were invited to complete a semi-structured interview to share their experiences.

### Data collection and rigour

Participants were interviewed individually on the final day they attended the clinic for the meal challenge or via telephone within one week of completing their final meal challenge. The interviews were designed to last approximately 30 minutes so that they could be conducted whilst visiting the lab for biological sample collection. To ensure participants’ experiences were explored in sufficient depth while maintaining commitment to the research aim, a series of questions were developed to guide the semi-structured interviews (Table 1). The question logic sought to elicit understanding of why people eat on night shift, strategies used by participants to avoid eating at night when participating in the intervention, explore the sensory elements of the workplace break environment, and understand motivation for participating.

**Table 1:** Open ended questions used to guide semi-structured interviews and associated logic.

Question	Logic
What are some of the foods you ate on night shifts and why?	Icebreaker- not data gathering
Tell me what it’s like working shift work?	Understand factors that might affect compliance to the protocol and sustainability of adherence, such as social and organisational factors
Tell me about what night shift was like on the days you avoided food intake?	Understand the nature of any physiological, emotional or psychosocial effects of the intervention
What are some of the things you did to help you change your eating times?	Understand enablers and strategies to maintain overnight food avoidance. This will inform what advice may be given to promote success in adhering to the protocol in the future
What are some of the reasons why you got involved in this study?	Explore effective ways to position/ pitch overnight food avoidance in a way that appeals to a shift worker audience. Understanding motivations for participating in the study may also provide insight into shift workers’ needs, such as health concerns
How would you feel about restricting your eating times long term? What should we consider to make this easier to follow in the future?	Understand the sustainability of overnight food avoidance and the role health professionals can play in facilitating and guiding workers through the process
Describe your workplace environment and what options you have for places to go on your break What foods/ smells/ cooking occurs in these spaces? How does it make you feel seeing/ smelling other people’s food during your shift?	Explore sensory elements of the workplace food environment and how they can be barriers or enablers. Understand how workplaces may be adapted to support workers in maintaining overnight food avoidance

One researcher who was familiar with the intervention study, but had no prior contact with participants, undertook all the interviews over the phone or in person. Interviews were audio-recorded and transcribed. Data were stored on a protected electronic laboratory notebook system (LabArchives, 2009). The interviewing researcher was introduced to participants by one of the researchers managing the intervention trial. The participants were made aware that the researcher conducting the interviews was a member of the research team and familiar with the study. To try to reduce bias, the interviewer reminded the participants to speak freely and honestly about their experience, as that helps to understand the feasibility of the intervention. The interviewing researcher has a background in nutrition and a mixed quantitative and qualitative research background.

### Data analysis

The data were analysed thematically with an inductive approach, allowing the data to be constructed into themes reflecting participants’ experiences. This was facilitated by an iterative process of coding, whereby the researcher assigned labels to interview data, reflected on similarities or differences between labels assigned to different interviews, and grouped labels if patterns emerged. Sub-themes were also annotated to indicate if the premise of the sub-theme reflected an enabling factor (+) or barrier (–) to participants during the intervention. The coding process was conducted on a data management software, NVivo (QSR International. NVivo for Macintosh. Version 12.0. Melbourne, VIC; 2018). The researcher kept a reflective journal on each interview after transcribing and coding, which encouraged reflexivity in the interpretation of interview data. A second researcher analysed all interview transcripts using the same approach. Consultation occurred between the researchers during the coding process to discuss interpretative differences and definitions of key codes, which were then developed into subthemes and themes. To synthesise the codes and aid the development of the final set of themes, the researchers collaboratively produced a mind map. Direct quotes from transcripts are provided as evidence and explanation of themes. After the thematic analysis was completed, the themes were mapped to the components of the Capability Opportunity Motivation-model of behaviour (COM-B)^24^ and to the domains of the Theoretical Domains Framework (TDF)^22^ to develop an understanding of how these data can inform implementation strategies for interventions targeting eating behaviours in night shift workers. The Theoretical Domains Framework was selected because it was developed to examine the influences on behaviour change and guide implementation design. It has a wide range of applications and considers contextual factors as well as individual behaviours^22^.

### Ethics approval

Ethical approval was granted by the Institutional Human Research Ethics committee (2017-8619-10329). Participants were provided with verbal and written statements of the study and gave written signed consent prior to participation. All methods were performed in accordance with The National Statement on Ethical Conduct in Human Research 2007 (updated 2018). (The National Health and Medical Research Council, the Australian Research Council and Universities Australia. Commonwealth of Australia, Canberra).

## Results

**Figure 1:**
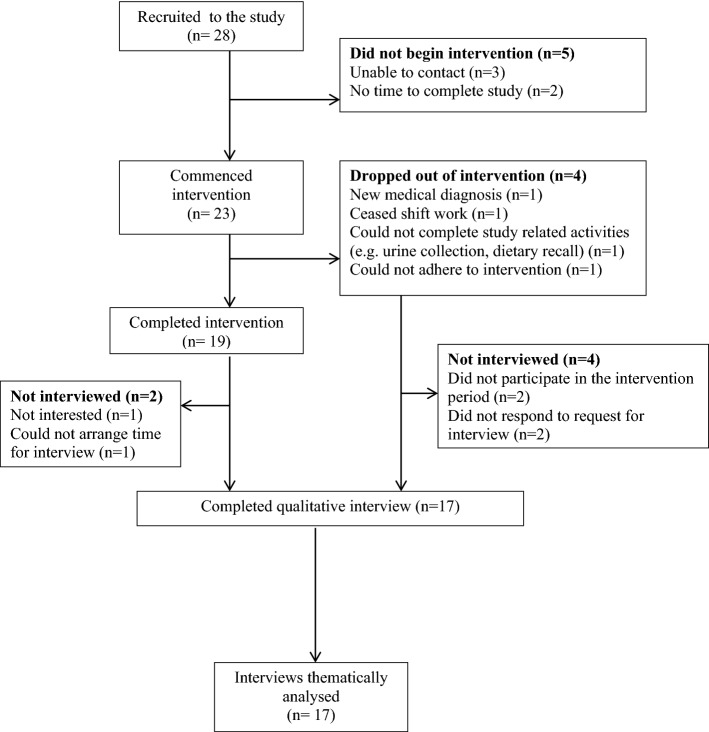
Flow diagram of participant recruitment to the qualitative evaluation.

Seventeen night shift workers (11 women and 6 men) were interviewed and interviews averaged 20 min in duration. The flow of participants in the intervention study is shown in Fig. 1. No interviews were held with participants who withdrew from the study (Fig. 1). Characteristics of the interviewed participants are summarised in Table 2; the majority of participants were overweight or obese and had been working shift work between 14 months and 28 years.

**Table 2:** Participant characteristics.

**Characteristics**	n (%)
**Sex**	
Male	6 (35)
Female	11 (65)
**Age (years)**	
25–35	5 (29)
36–45	5 (29)
46–55	6 (35)
56–65	1 (6)
**Body mass index (kg/m**^**2**^**)**	
Healthy weight 18.5–24.9	1 (6)
Overweight 25–29.9	9 (53)
Obese ≥ 30	7 (41)
**Occupational field**	
Health professionals	9 (53)
Hospitality and food services	4 (24)
Public transport services	1 (6)
Labourers and factory workers	3 (18)

From the analysis of participants’ experience with the meal timing intervention, fourteen subthemes were revealed and combined into three major themes: ‘the burden of shift work leads to food temptations’; ‘workplace structures, environment and culture’; and ‘willingness to change requires individual motivation.’ (Table 3).

**Table 3:** Thematic representation of shift workers’ experiences and perceived feasibility of a meal timing intervention during night shift.

Themes	Sub themes + perceived facilitators − perceived barriers		Related domains of the Theoretical Domains Framework (TDF)	COM-B Components
The burden of shift work leads to food temptation	Keeping busy	+	Environmental context Behavioural regulation	O C
	Water consumption/ importance of feeling full	+	Behavioural regulation	C
	Comfort eating	−	Emotion	M
	Caffeine	–	Behavioural regulation Emotion	C M
	Physical and mental stress	–	Emotion Beliefs about capabilities	M M
	Co-worker support/social influences	+ /−	Social influences	O
	Break environments	–	Environmental context	O
Workplace structures, environment and culture	Meal break timing (organisation determined)	+ /–	Environmental context	O
	Restructured eating schedule (individually determined)	+ /–	Environmental context	O
	Management influences	+	Environmental context	O
Willingness to change requires individual motivation	Education	+	Knowledge Social influences	C O
	Mental determination	+	Beliefs about capabilities	M
	Body weight	+	Reinforcement	M
	Health beliefs	+	Emotion Beliefs and consequences	M M

Themes and subthemes are linked to the Theoretical Domains Framework (TDF)^22^and the behaviour change model constructs: Capability, Opportunity and Motivation (COM-B)^24^. + indicates the subtheme was reported as an enabler to following the intervention;—indicates the subtheme was reported as a barrier to following; + /– indicates the subtheme was reported as both an enabler and a barrier to following the intervention. Some sub-themes were mapped to more than one domain of the TDF. Two subthemes by the broken lines were common to two of the major themes. C, capability, O opportunity, M motivation.

### The burden of shift work leads to food temptations

People spoke extensively about the fatigue and sleepiness they experienced as a result of doing night shift work. Initially some participants felt that avoiding eating between 1 and 6am would be both physically and mentally challenging, as eating helped them get through their strenuous night shifts. This mindset subsided throughout the intervention, with the realisation that food was not as vital to their mental endurance as they had originally perceived. One participant who reported that their main reason for eating on night shift was to ‘*be more mentally alert*’ *(P05),* ultimately concluded that after the intervention, they actually ‘*didn’t notice much difference in [their] alertness*.’ *(P05).*

Avoiding caffeine rather than food was viewed as a bigger challenge of the intervention. Almost all participants expressed their desire to have tea or coffee over the fasting period. Caffeine was perceived as important to help overcome the fatigue and sleepiness that accompanies night shift work. It was seen as a reprieve from the stresses of night shift work, and acted as a ‘*relaxant*.’ *(P05).*

My coffee and my green tea… I can pretty much do without food, but I need my coffee and my green tea. P06

Participants faced a number of food temptations during the four week intervention period, driven by social influences, the meal break environment, boredom, cravings, and a desire for comfort. Some strategies adopted during the fasting period were specifically aimed at mitigating the effects of these temptation factors. Maintaining a feeling of ‘fullness’ during the fasting period was a crucial strategy reported by the majority of participants. Increased water consumption was how participants curbed feelings of hunger to create a feeling of fullness.

The participants described how they and their colleagues commonly consumed food as a source of comfort for the various hardships associated with night shift work, creating an emotional attachment to food. Social eating formed a major part of shift work culture. This was particularly evident in nursing environments, where participants described their team of colleagues as ‘*family*’ (P13). Workers commonly brought food to work to ‘*treat’* or *‘spoil’* (P08) their colleagues. For many, it is common practice to bring food to work for sharing as it creates a ‘*party every night*’ (P08). This is distinct from when they worked their day shifts, where bringing in food to share was less common.

It must be like a mindset on night duty… because we have to do it, we have to reward ourselves and that’s what we do, that’s how we reward ourselves. That’s how we lure ourselves to be there [on night shift] or something. (P13)

One participant described a cook-up that their ‘*warm and welcoming*’ (P01) colleague routinely put on during night shift, emphasising that food positively affects the mood of workers—*'… She does a lot of comfort food, which is just lovely. It’s just lovely. And it’s morale boosting…'* (P01)

There was a dearth of strategies adopted by participants for overcoming the loss of social connectedness that eating occasions usually provided. The participants in our study were highly engaged and motivated, demonstrating a strong commitment to the research study and reported being able to easily refuse offers of food from colleagues during the intervention period.

They would have stuff out and you know, ‘Oh, my partner made brownies… do you want some?’ And I’d say, ‘Look, I can’t, I’m not allowed to.’ So yeah, I was happy to do that. (P13)

### Workplace environment, structures and culture

Workplace characteristics were heterogeneous amongst the participants, which influenced participants’ experiences of the intervention including, meal break scheduling, and supportive management and co-workers. Participants described how being surrounded by the smells, sights and sounds of food in break areas were some of the challenges they had to overcome to adhere to the intervention of avoiding eating between 1 and 6 am. Break environments were commonly centred on food and eating, with few accessible areas to retreat away from food at night.

There are quiet rooms, but they’re… I find them a bit cold. And not inviting, to be honest. There’s a room downstairs, but again, you’ve got to go down into a, you know, down a creepy stairwell and into the lower level… Some people go outside and go for a walk around the carpark… I certainly would not be going outside on my own you know, in the middle of the night. (P01)

Workers who did find ways to avoid eating environments during their break used a number of unique strategies, but these were not satisfactory or sustainable.

I spent a bit of time in like the women’s toilets at my work have like a couch and like a little quiet room that you can sleep in a stuff so I’d go and sit in there alone. (P12).

Others stayed at their desks, or found nooks in their workplaces that were away from food temptations. Although participants found these particular rest areas aided them during the intervention, none of the break environments that were described represented an ideal, comfortable, communal space that was free from any association with food. Participants expressed a desire for more pleasant spaces, claiming it would help with adhering to the intervention over the long term. Furthermore, they also felt they had little control over making this a reality, believing that those in ‘higher-up’ positions held this power. The consequences of unsatisfactory break environments free from food resulted in participants experiencing a stronger desire to eat. Shifting focus towards their phones or the television during work breaks was a helpful strategy to overcome feelings of hunger and food temptations. Participants described how work responsibilities helped to keep them distracted from the thought of eating. Many committed themselves to working over the fasting period, with some even shortening their breaks if they fell within the fasting period of 1am and 6am. This is not a sustainable practice for long term adherence to the intervention, and will require changes to workplaces’ physical environments to support workers.

I might have just gone out… then gone straight back sort of 10 min later rather than have my full allotted half hour. (P11)

When able to, participants planned their meal breaks to occur prior to the fasting period. Participants claimed this helped them feel fuller to *‘get through’* (P08). Not all participants had the flexibility to choose when their breaks were scheduled. In other situations, breaks did not occur at the planned time because of unexpected clashes with work responsibilities, which took priority over a break.

There were shifts where we’d get an admission right on 12 o’clock. And so my plan would go out the window. (P08)

The strictness of the timing of the fasting period (1–6 am) created a challenge for those who did not have flexibility over their break schedules. This highlights the need for interventions to be flexible or adaptable to suit different workplace schedules.

Because the meal breaks were sporadically designated, we couldn’t always organise for myself to have a meal break anywhere between midnight and 1am. So sometimes I have to take a quick, you know, sort of 5–10 min and smash down [food]. (P11)

A strong emphasis on staff health and wellbeing was evident in some participants’ workplaces, a testament to the leadership within these workplaces in shaping the health culture. A strong health culture, backed by staff at the management level, was suggested to be an enabler to adhering to the intervention. Supportive colleagues reminded participants to avoid eating between 1 and 6am and helped them to coordinate meal breaks around this period. These social supports were more evident in team-based work environments, and less so in independent work environments.

One of the shifts I was right on like five to one, and I’ve looked at the clock and I’ve looked at my coworker who was with me most shifts, and I’ve looked at her and I’ve gone, ‘I’ve got to go and eat!’ And she’s like ‘Get out of here! Go!’ And she just pushed me out of the room, took over what I was doing with the admission and sent me to go and eat because she knew that after 1 o’clock I was done. (P08)

### Willingness to change requires individual motivation

Participants commonly spoke about being overweight and attributed their weight gain to night shift work. There was a belief amongst some participants that timing of meals and snacks was a cause of the weight gain.

Everyone now, like that I speak to in the industry, they say, you know, they talk about weight and whatever, and they go, "It’s because we have a big meal and then we go to bed. (P02)

The majority of night shift workers reflected that adhering to the intervention was feasible with a disciplined and determined mindset. It was also commonly reported that this mindset took a while to develop, but eventually the intervention became easier to comply with. The desire to lose weight was a factor in many participant’s motivation to take part in the intervention study, despite being made aware that this intervention was targeting metabolic changes (e.g. blood glucose and blood lipids) and not weight loss. When we asked the participants about their intention to continue with the meal timing intervention after study completion, it was commonly stated that because they could not see any physical changes to their health or weight, their motivation to continue was low.

Participants indicated that willpower, rather than education, was the key to inciting and maintaining behaviour change. Some participants suggested that night shift workers know how to be healthy, but generally lacked the determination to follow through with this knowledge.

I think… there has to be a ‘what’s in it.’ For people to look at a food study… Because I have put on weight [due to shift work]. I know that I’m not managing my food intake as well as I could be. (P01)

When reflecting on the long-term feasibility, participants expressed that greater awareness of the risks of eating at night and the benefits of an overnight fast requires widespread promotion to not only night shift workers but the general community. There was a belief that greater awareness would lead to greater support for shift workers to achieve behaviour change.

Just educating people more about… the benefits to having that period of fasting after midnight… you know, you don’t normally eat after midnight, so think about what you’re doing. (P01)

‘In my workforce, nothing you could do would change their behaviour… probably public knowledge so their partners or their significant others could understand the importance of changing dietary habits at night.’ (P05).

## Discussion

The feasibility of this simple meal timing intervention to avoid eating between 1 and 6 am on night shift was largely affected by three major influences (1) physical and emotional burden of shift work which drives food temptations; (2) the workplace context including the meal break environment, social and cultural context at work, and break scheduling; and (3) motivation of the individual. Through mapping of these findings to the Theoretical Domains Framework and Capability Opportunity Motivation model of behaviour (COM-B)^22,24^, we identify the influences on behaviours that can be targeted to further support night shift workers to follow this intervention.

Night shift workers have a social and emotional connection to food at night^19,25^. Our participants who worked in team environments, particularly in nursing, described that night shift created a deep sense of comradeship, even kinship, with their co-workers. Food played an important role in these relationships, to celebrate or reward themselves for working during the night. For others, eating throughout the night shift was a form of comfort. To overcome these food temptations during the intervention, participants required mental determination and motivation. Bonnell et al.^19^ found that communal eating amongst night shift-working firefighters was common, and postulated that this ‘group culture’ is particularly poignant in workplaces where a sense of collegiality and teamwork is integral to performance in demanding work conditions. Although some participants strongly emphasised that social influences drove them to eat at night, others also reported that supportive colleagues helped them to adhere to the intervention. The social cognitive theory explains how a socially orientated approach to health leads to favourable and enduring behaviour change of individuals^26^. For individuals to have control over their health and health behaviours they need the collective support of their community. Other studies of shift-working populations have demonstrated that the ‘group culture’ phenomenon encourages both positive and negative health behaviours^19,25^. The PHLAME firefighters study was a 12 month randomised controlled trial of 599 firefighters participating in a healthy lifestyle promoting program^27^. Elliot et al. (2004)^27^ demonstrated that in a team-based work environment, adoption of health behaviours is feasible and leads to a reduction in disease risk markers (attenuated weight gain and increased fruit and vegetable intake).

Participants explained how ongoing motivation to follow dietary interventions would be augmented by evidence that their efforts are having benefit on their health. These findings may be explained by the Health Belief Model, which indicates that one of the key criteria people use to make an assessment of whether to commit to a health strategy is ‘perceived benefits of behaviour change’^28^. The outcomes targeted in this meal time intervention were metabolic changes (i.e. blood glucose and blood lipids) that are not easily monitored by participants. Participants expressed major concerns about weight gain and attributed this to shift work, suggesting that interventions targeting weight loss may be important to this population. Another construct of the Health Belief Model, ‘cues to action,’ would be necessary to consider as this alerts to factors which trigger behaviour change, and education is a key example of such a factor^29,30^. Participants in this study believed that educating night shift workers about the benefits of overnight food avoidance would be an enabler to undertaking and maintaining the intervention. Participants suggested that educating family members of shift workers could also be an effective strategy, to incite behaviour change in shift workers who are not self-motivated or have little concern for their own health. Previous research has demonstrated that social support, particularly from family members, is a valuable approach to encourage the uptake and sustainability of health interventions^31,32^. In the context of shift work motivation for behaviour change is harder to achieve, and support and education alone may be inadequate. In a cross-sectional study of 1390 overweight shift workers, motivation for a behavioural change in lifestyle was lower compared with overweight day workers (n = 7853)^33^. Shift workers experience high levels of work-related stress that negatively affects motivation to change lifestyle behaviours^34^ and can lead to weight gain^35^. A study to increase physical activity levels of nurses (n = 70) found that work-related factors (e.g. shift schedule and role type) predicted the change in moderate physical activity achieved^36^. Interventions to support lifestyle behaviour change need to be multicomponent to address causes of workplace stressors^36,37^ additional to behavioural-based interventions aimed at mitigating the neuroendocrine abnormalities of circadian disruption^9–16,38^.

The workplace context was identified as both a facilitator and barrier to supporting our participants to follow the nutrition intervention. This included the physical environment, organisational culture and shift structure (e.g. meal break schedules). Previous studies have reported that eating on night shift is affected by shift schedule structures which can lead to eating despite not being hungry^38,39^. A study of student nurses found that the workplace exposed them to high levels of unhealthy food and alcohol behaviours and identified improving culture and leadership as a strategy for supporting them to adopt healthier behaviours^40^. A review of factors affecting healthy eating found that work factors including long work hours, shift work, a high workload and low staffing levels were barriers to healthy eating^41^ indicating that organisational leadership is required to change these structures. In the present study, to facilitate adherence to the intervention, participants indicated that planning their meals prior to, and during, shift was important which is consistent with other studies that have reported time for planning meals is a facilitator of healthy eating behaviours^42,43^. When workplaces adopt a culture of healthy lifestyle behaviours, people are more encouraged to allow time to plan for eating healthier at work.^43^ and adopting health promoting behaviours in general^44^. Our participants who did not have access to these enabling factors, resorted to unsustainable practices including working through their break periods. Engagement in work activities during break times has been associated with poorer work performance and general wellbeing^45^. Employers could consider spaces that promote physical activity during night shift, as this has been associated with improved well-being and performance^27,46^.

### Strengths and limitations

A strength of the study is that participants came from a diverse range of occupations and industries, which provided a rich insight into the influences of workplace characteristics. A further strength of this study was that data saturation was achieved following the transcription of all interviews. Therefore, this paper provides a comprehensive overview of participants’ experiences of the Shifting the Risk Study. This study has a few limitations. The participants in this study may represent a selected population of night shift workers who were motivated to participate because of their beliefs that night shift work has negative effects on their health, such as weight gain. Participants included a mix of permanent and rotating night shift workers and it’s possible that the experiences of adhering to a meal timing intervention are different for these different working groups which was not captured in this analysis. Only participants who had completed the intervention consented to an interview. Those who dropped out were invited for an interview but declined. These participants could potentially provide alternative perspectives on the feasibility of the intervention, which have not been covered here. This study was conducted in one country and may not be generalisable to night shift workers in other countries.

## Conclusion

The intervention study asked participants to avoid eating between 1 and 6 am for four weeks. This qualitative exploration identified that key facilitators to avoiding eating at night were; keeping busy, having co-worker support, management support, education of health benefits and/or belief that the intervention was health promoting. The barriers to avoiding eating at night were the emotional and physical toll of working at night leading to comfort eating and not having rest areas away from food environments. From night shift workers’ perspectives, the sharing of food provides a social and emotional response, which helps them to endure the tolls of night shift work. This study found that workplaces can support individuals in choosing health promoting behaviours on night shift by making changes to the workplace culture and environment including: permitting flexible break times, and providing spaces free from food and away from work duties that enables social connectedness. Future studies investigating meal interventions for night shift workers should consider the changes needed in the workplace environment to support implementation.

## Data Availability

The datasets generated and/or analysed during the current study are not publicly available as ethical approval to share the individual level data was not obtained. Approval was only granted to share aggregate level data to protect the privacy and confidentiality of participants as the qualitative data records contain re-identifiable data.
